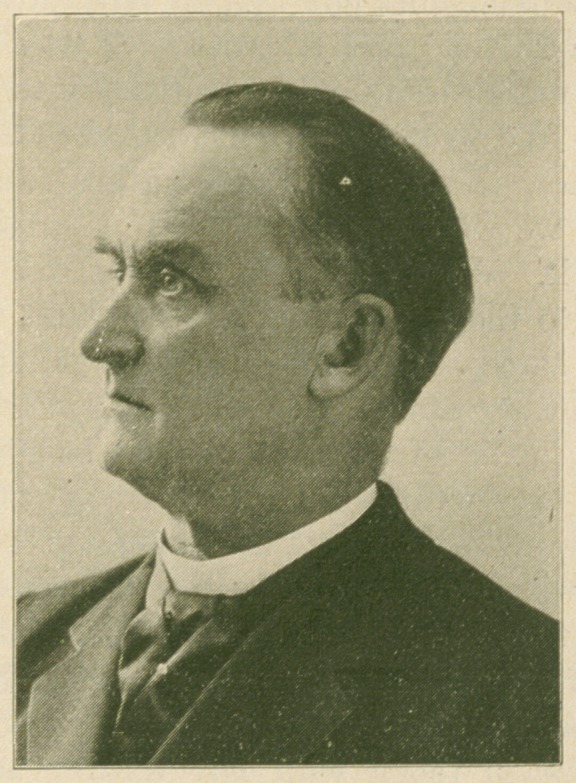# David C. Whaley, D.D.S.

**Published:** 1913-02-15

**Authors:** 


### OBITUARY.

### DAVID C. WHALEY, D.D.S.

Dr. Whaley, probably the oldest dental practitioner in Southeastern Ohio died at his home in Pomeroy, O., Dec. 30th, 1912, in his 85th year. Dr. Whaley practiced dentistry in Pomeroy, Ohio, continuously for over fifty years. He was well-known throughout that section of the country as a most skillful practitioner, consequently he had always a large and remunerative practice. He studied dentistry first with a physician having previously learned the cabinet trade at which he was very skillful. He attended the Ohio College of Dental Surgery afterwards, from which school he was graduated in 1865. He was a man of strong character and exerted a good influence in the community where he lived. He was interested in all civic affairs and while never a politician, he always used his influence and gave of his time and resources in behalf of clean government and good morals. He was not a contributor to his professions literature, nor an active participant in its general activities, yet he kept himself posted as he always read the professional literature and attended the State meetings frequently. He was a thorough and conscientious workman and all his work was done with care and an unusual degree of proficiency. He was a man of strong convictions and many of his methods of practice he worked out and confirmed in his own experience and it was interesting indeed to hear him discuss methods of practice with a practitioner who differed with him. He always knew why he used a method and was not easily persuaded to adopt methods that would not stand the test of practical experience. He had a strong constitution and an energetic habit that enabled him to do an extraordinary amount of work. He applied himself so diligently and faithfully to his practice that he accumulated considerable property. The community which he has served so long and so faithfully owes him a debt of gratitude of which it has no adequate conception, and the thousands of patients to whom he has ministered ought to remember him and his faithful work in their behalf with gratitude and affection. It was Dr. Whaley who put the notion of studying dentistry into the mind of your editor. We went to his office to have a tooth filled (the first one), shortly after graduating from the high school, when we were trying to select a suitable vocation for life's work. We had turned down a call to medicine and another to the ministry, and were about to engage in a mercantile career. Our appointment was interrupted by a call from a lady coming from a long distance out in the country for a full set of teeth, which must be gotten out that day and the lady went home that evening with her teeth, and we helped make them by watching the process and lending a hand wherever we could. We got so interested in the work that we spent three or four days with the Doctor and got all of our teeth fixed up and incidentally were stung with the dental microbe, from which we have never recovered. We have kept in touch with Dr. Whaley and his work ever since, and we shall always feel grateful for his directing us in the right path at a time when we were almost at sea. Our friendship has been constant throughout all these years and we hope to carry to eternity the first filling we ever had a mesial-proximal in the left upper lateral. So far as we can determine after 40 years of hard usage it seems destined to last as long as we shall need it. If our profession had more such conscientious and capable practitioners our calling would be better and more honored. We shall miss him.

## Figures and Tables

**Figure f1:**